# Iron Accumulates in Huntington’s Disease Neurons: Protection by Deferoxamine 

**DOI:** 10.1371/journal.pone.0077023

**Published:** 2013-10-11

**Authors:** Jianfang Chen, Eileen Marks, Barry Lai, Zhaojie Zhang, James A. Duce, Linh Q. Lam, Irene Volitakis, Ashley I. Bush, Steven Hersch, Jonathan H. Fox

**Affiliations:** 1 Department of Veterinary Sciences and Neuroscience Graduate Program, University of Wyoming, Laramie, Wyoming, United States of America; 2 Argonne National Laboratory, Lemont, Illinois, United States of America; 3 Department of Zoology and Physiology, University of Wyoming, Laramie, Wyoming, United States of America; 4 Mental Health Research Institute, Parkville, Melbourne, Victoria, Australia; 5 School of Molecular and Cellular Biology, the Faculty of Biological Sciences, University of Leeds, Leeds, United Kingdom; 6 MassGeneral Institute for Neurodegenerative Disease, Charlestown, Massachusetts, United States of America; Alexander Fleming Biomedical Sciences Research Center, Greece

## Abstract

Huntington’s disease (HD) is a progressive neurodegenerative disorder caused by a polyglutamine-encoding CAG expansion in the huntingtin gene. Iron accumulates in the brains of HD patients and mouse disease models. However, the cellular and subcellular sites of iron accumulation, as well as significance to disease progression are not well understood. We used independent approaches to investigate the location of brain iron accumulation. In R6/2 HD mouse brain, synchotron x-ray fluorescence analysis revealed iron accumulation as discrete puncta in the perinuclear cytoplasm of striatal neurons. Further, perfusion Turnbull’s staining for ferrous iron (II) combined with transmission electron microscope ultra-structural analysis revealed increased staining in membrane bound peri-nuclear vesicles in R6/2 HD striatal neurons. Analysis of iron homeostatic proteins in R6/2 HD mice revealed decreased levels of the iron response proteins (IRPs 1 and 2) and accordingly decreased expression of iron uptake transferrin receptor (TfR) and increased levels of neuronal iron export protein ferroportin (FPN). Finally, we show that intra-ventricular delivery of the iron chelator deferoxamine results in an improvement of the motor phenotype in R6/2 HD mice. Our data supports accumulation of redox-active ferrous iron in the endocytic / lysosomal compartment in mouse HD neurons. Expression changes of IRPs, TfR and FPN are consistent with a compensatory response to an increased intra-neuronal labile iron pool leading to increased susceptibility to iron-associated oxidative stress. These findings, together with protection by deferoxamine, support a potentiating role of neuronal iron accumulation in HD.

## Introduction

Huntington’s disease (HD) is a progressive neurodegenerative disorder characterized by motor, psychiatric and cognitive disturbances that progresses to dementia [1]. Prevalence is Europe, North America and Australia is ~ 5.70 per 100,000 [2]. HD is caused by a dominant CAG expansion in the exon-1 encoded region of the huntingtin gene resulting in the expression of polyglutamine-expanded mutant huntingtin protein (mhtt) [1]. HD brain regions (especially striatum and cerebral cortex) undergo progressive degeneration starting several years before clinical onset [3]. Numerous mechanisms have been implicated in the pathogenesis of HD including oxidative stress [4], energetic dysfunction [5,6], transcriptional dysregulation [7,8] and defective axonal transport [9]. However, despite this, there are currently no protective therapies for human HD. Central nervous system (CNS) iron dysregulation occurs in human HD [10,11] and also other neurodegenerative diseases including Alzheimer’s (AD) [12], Parkinson’s [13,14], Lou Gehrig’s disease [15], prionopathies [16], neuroferritinopathy [17] and aceruloplasminemia [18]. Importantly, HD brain magnetic resonance imaging of gene-positive individuals has shown that alterations of brain iron homeostasis occur before the onset of clinical signs [10] suggesting an early role in the disease process. However, whether changes in brain iron contribute to disease onset / progression, represent a protective response or arise as an epiphenomenon is not fully understood. 

Huntingtin protein (htt) is implicated in cellular iron homeostasis. Huntingtin knockdown in zebra fish results in an iron deficiency phenotype [19]. Conversely, mhtt expression results in brain iron accumulation in human HD [10,11]. Htt level and / or glutamine expansion within htt is therefore an important modulator of iron status. We have shown that iron does not interact directly with N-terminal htt fragments [20] indicating that htt’s effect on iron are mediated by downstream influences on iron homeostatic pathways. Several iron homeostatic proteins are involved in neurodegenerative disease processes [21-23]. For example, amyloid precursor protein (APP) facilitates neuronal iron export [22] and in brain is expressed essentially in neurons [24]. Reduced expression of APP or impaired trafficking to its correct location on the cell surface correlates with brain iron elevation in mouse models of AD [21,22]. Mutations of the ceruloplasmin gene, which encodes a glial expressed ferroxidase also results in brain iron accumulation leading to degeneration primarily within the striatum; a vulnerable region in HD [18,25]. As the cell is susceptible to changes in iron homeostasis, its intracellular status is very tightly regulated by iron-response proteins 1 and 2 (IRP1 and IRP2). By monitoring the labile iron pool within the cell, IRPs control the translation of proteins responsible for iron uptake (e.g. transferrin receptor; TfR), intracellular storage (e.g. ferritin; Ft) and export (e.g. ferroportin; Fpn [26]). Investigating these proteins can provide an important indication of functional iron status (e.g. deficiency or toxicity) in different disease states. 

Numerous genetic mouse models of HD accurately recapitulate many of the features of human HD including neurodegeneration of striatum and cerebral cortex [27,28]. We have previously reported elevation of brain iron in two mouse HD models [20], demonstrating that HD mice provide a valuable system for investigating iron dysregulation in human HD. Iron is instrumental in numerous heme and iron-sulfur proteins [14] and can also exist in an unbound form able to generate reactive oxygen species leading to neurotoxicity [12]. Therefore, understanding the cellular and subcellular sites of iron accumulation, as well as molecular associations, is essential to understanding its role in diseases such as HD.

The aim of this study was to investigate whether brain iron elevation contributes to disease pathogenesis in a mouse model of HD. We focused on identification of the cellular and subcellular sites of iron accumulation, characterization of changes in iron protein homeostatic machinery and effects of an iron modulatory treatment. Our findings support a potentiating role of elevated neuronal iron in disease pathogenesis and indicate that treatments that decrease neuronal iron may provide protection in human HD.

## Materials and Methods

All mouse experiments were approved by the University of Wyoming and MassGeneral Institute for Neurodegenerative Disease Institutional Animal Care and Use Committees and were also in accordance with NIH guidelines. 

### Supplies

Four primary antibodies were used. The polyclonal goat anti-mouse transferrin (1-20) was purchased from Santa Cruz Biotechnology, Inc.. Monoclonal mouse anti-transferrin receptor and anti-mouse ferritin were supplied by Invitrogen while monoclonal anti-mouse TfR, anti-mouse IRP-1, anti-mouse IRP-2 and polyclonal rabbit anti-ferroportin were from Alpha Diagnostics International. Actin antibody (AC40) was from Sigma. HRP conjugated secondary antibodies were from Abcam. Fluorescent secondary antibodies were from Invitrogen. All chemicals were from Sigma.

### Mouse husbandry

R6/2 and N171-82Q HD mice were maintained by backcrossing HD males with F1 females of the B6/CBA or B6/C3H backgrounds, respectively. Tail tips cut at 3 weeks of age were used for genotyping as described previously [29]. Mice were grouped by genotype or treatment group under standard conditions with a 12 hour dark-light cycle. CAG repeat sizes for R6/2 and N171-82 HD mice averaged ~181 and 82, respectively. 

### Mouse behavioral analyses

For accelerating rota-rod analysis we used the instrument from IITC Life Science which has a 1.25 inch diameter drum and was set to have linear acceleration over a 15 minute period from 5 to 45 revolutions per minute. For each time point, mice were given one training session. They were then tested once a day over the next three consecutive days during the dark phase. For in-cage spontaneous wheel running we used the wireless wheel running system from Med Associates, Inc. For each time point, mice were housed individually in cages for 4 days. Data was recorded on days 2-4. 

### Stereotaxic surgery

Subcutaneous Alzet mini-osmotic pumps (model 1002) were implanted subcutaneously with an intra-ventricular cannula (Alzet brain infusion kit) placed into the left lateral ventricle at the following stereotaxic coordinates with respect to bregma (lateral: 1.0 mm, deep: 2.5 mm, rostral: 0.14 mm). Mice were anesthetized with ketamine / xylazine. 

### Neuropathology

Mice were perfused through the left ventricle with 4% paraformaldehyde. Brains were removed, immersion fixed for a further 24 hours then placed in cryopreservant (10% glycerol, 2% DMSO and 0.1 M phosphate buffer (pH 7.4)) for 3 days prior to serial sectioning at 50 µm. The Cavalieri method was used to estimate ventricular volumes using the StereoInvestigator® stereologic system.

### Inductively-coupled-plasma mass spectroscopy (ICP-MS)

Mice were anesthetized with ketamine/xylazine then perfused with 0.9% (w/v) saline containing 25 units / ml heparin for 2 minutes at a rate of 10 milliliters (mls) per minute. Striatum and cortices were dissected, frozen immediately on dry ice and stored at -80°C. Dissected brain regions were later weighed, lyophilized then analyzed for iron by ICP-MS exactly as described previously [30].

### Non-heme iron analysis

Preparation of tissue was as for ICP-MS. The assay uses acid hydrolysis to release non-heme iron from protein. Iron (III) is then reduced to iron (II) which is quantified with bathophenanthroline by absorption at 535 nm. In brief, brain tissue was homogenized in 14 volumes of RIPA buffer (PBS containing 1% IPEGAL, 0.5% sodium deoxycholate and 0.1% SDS) then centrifuged at 16 000g for 10 minutes. To 100 µl of supernatant 50 µl of acid was added (25.9% HCl and 30% (w/v) trichloroacetic acid). The sample was heated to 65°C for 18 hours with shaking. The sample was then centrifuged and aliquots of supernatant used for determinations as described [31]. Quantification utilized iron standards and buffer blanks. 

### Synchotron x-ray fluorescence

Mice were sacrificed by barbiturate overdose. Brains were removed and frozen in liquid nitrogen vapor. Ten µm sections were cut on a cryostat, mounted onto silicon nitride membrane then lyophilized for 30 minutes. The hard x-ray microprobe is located in a dedicated beamline (2-ID-D) that was specifically developed for x-ray microscopic applications. This technique enables determination of trace element distribution at the sub-cellular level. The detection limit for iron and zinc is ^≈^10^-19^ mol·μm^-2^ Recent advances of high-brilliance synchrotron radiation sources and x-ray Fresnel zone plate microfocusing optics with high spatial resolution and high focusing efficiency allowed the incident x-ray beam to be focused to a spot size of 200 nm. Lyophilized brain samples were raster scanned across the focus spot while the x-ray fluorescence spectrum from each pixel was recorded by an energy dispersive detector. Iron and zinc spectra were recorded. The method is described in detail in the supporting information. 

### Perfusion Perl’s and Turnbull’s staining of iron

Total iron was stained as previously described [22] using modified Perl’s staining that histologically detects all forms of iron; this was combined with bright field microscopic analysis. Specific forms of iron were stained either with unmodified Perl’s stain solution that reacts with available ferric iron (III), or Turnbull’s stain to detect ferrous iron (II). The method we used is a modification of that already described [32]. Mice were perfused with a solution of fixative then the Perl’s or Turnbull’s staining solution. This approach was combined with ultra-structural analysis and allows subcellular localization as well as estimate of the ionic state of available iron in brain tissue in-vivo. The method is described in detail in the supporting information.

### Western blot analysis

Perfusion and preparation of tissue was as for ICP-MS and the non-heme iron assay. Weighed brain regions were homogenized in 15 volumes of cell lysis buffer (20mM TRIS, 150mM NaCl, 1mM EDTA, 1% Triton X, pH 7.5) containing a protease inhibitor cocktail (Roche). Frozen samples were homogenized with a handheld homogenizer, centrifuged at 15000 g for 15 minutes then aliquots of supernatant stored at -80°C. Thirty µg of protein was separated by reducing SDS-PAGE using 4-12% gradient gels then transferred to PVDF. Primary antibody concentrations were DMT1 (1:200), transferrin (1:400), transferrin receptor (Invitrogen; 1:500, Alpha Diagnostic; 1:1000) ferritin (1:5000), IRP-1 (1:500) and IRP-2 (1:500). HRP labeled secondary antibodies were used at 1:2000 dilution. 

### Immunofluorescence

Mice were anesthetized, perfused via the left ventricle for 2 minutes with heparinized saline followed by fresh 4% paraformaldehyde in 0.1 M phosphate buffer (pH 7.4). Brains were removed then post-fixed overnight at 4° C prior. They were then cryopreserved in 10% glycerol, 2% DMSO and 0.1 M phosphate buffer (pH 7.4) at 4° C for >4 days prior to preparation of 40 µm sections using a freezing microtome. Sections at the level of the anterior commissure were probed with primary antibody at 4°C for 48 hours (1:200 dilutions for all antibodies used, diluted in PBS-0.1% tween-10% goat or rabbit serum). Sections were washed twice for 10 minutes in PBS, then placed in secondary antibody overnight at 4°C (1:500 dilution). They were then washed in PBS thrice for 10 minutes then incubated in the nucleic acid stain DRAQ5^TM^ (Biostatus Ltd.) at 20 µM for 30 minutes at 25° C. Following two washes in PBS they were mounted using Fluoromount G (SouthernBiotech). Blocking peptides as well as no primary and no secondary controls were used to determine the specificity of fluorescence signal. A Zeiss 710 confocal microscope was used to collect z-stacks in medial and lateral cerebral cortex (layer VI) and dorsomedial and midlateral striatum both at the level of the anterior commissure. For each z-stack, images 9 µm either side the center of the section were analyzed using Image J software. An image sequence was opened containing separate images of protein and DRAQ5^TM^ immunofluorescence. A circle was placed around nuclei to include cytoplasm, this was then transferred to the protein fluorescence image where the mean number of intracellular pixels within each tracing was measured.

### Statistical analyses

Data was analyzed by a student’s two-sided t-test, two-way ANOVA or repeated-measures ANOVA, as appropriate, using SAS software. Graphs represent means ± SEM. P-values <0.05 were considered significant. For each experiment ‘n’ is defined as the number of animals in each experimental group. 

## Results

**Figure 1:**
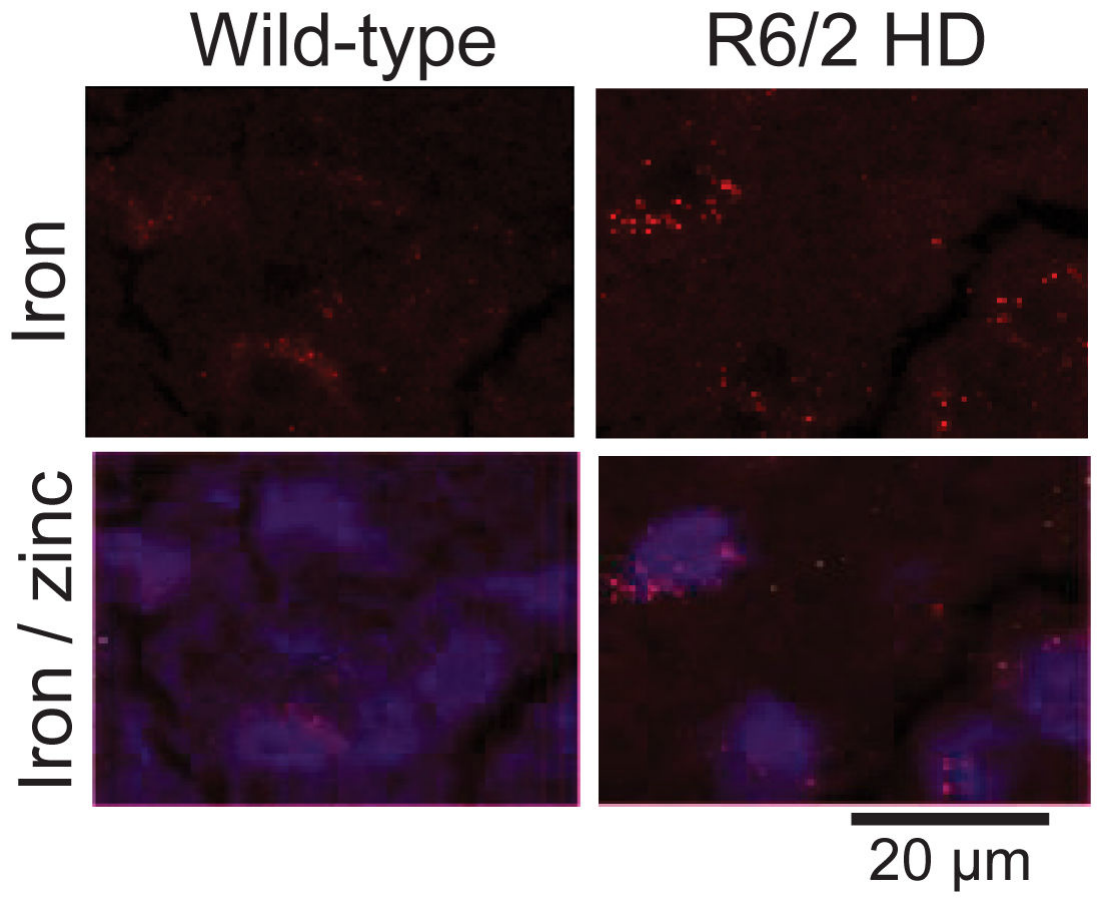
Iron accumulates in R6/2 HD striatal neurons. Striata of 12-week-old R6/2 HD and wild-type litter mate mice were studied by synchrotron x-ray fluorescence (XRF - see methods). XRF identifies neuronal nuclei due to high zinc content (blue) [33]. HD mice demonstrate punctate peri-nuclear (cytoplasmic) accumulations of iron. Shown are representative images. n=2.

R6/2 HD mice had elevations of cortical iron occurring around the time of onset of behavioral declines, while striatal iron elevation occured later (Figure **S1**). N171-82Q HD had elevations of cortical but not striatal iron (Figure **S2**). We investigated site(s) of brain iron elevation in R6/2 HD mice as these more closely resemble the human brain iron phenotype than N171-82Q mice [10]. Striata were studied due to greater tissue homogeneity as compared to cerebral cortex. Mice were 12-weeks of age at the time of analysis corresponding to late-stage disease in HD littermates. X-ray fluorescence (XRF) on lyophilized brain sections revealed neuronal nuclei due to high zinc content [33]. This allowed for neuronal identification / orientation within the striatal iron map. Iron fluorescence was detected as numerous small puncta of fluorescence in the perinuclear cytoplasm of striatal neuronal cell bodies as compared to wild-type litter-mate mice (Figure **1**). 

**Figure 2:**
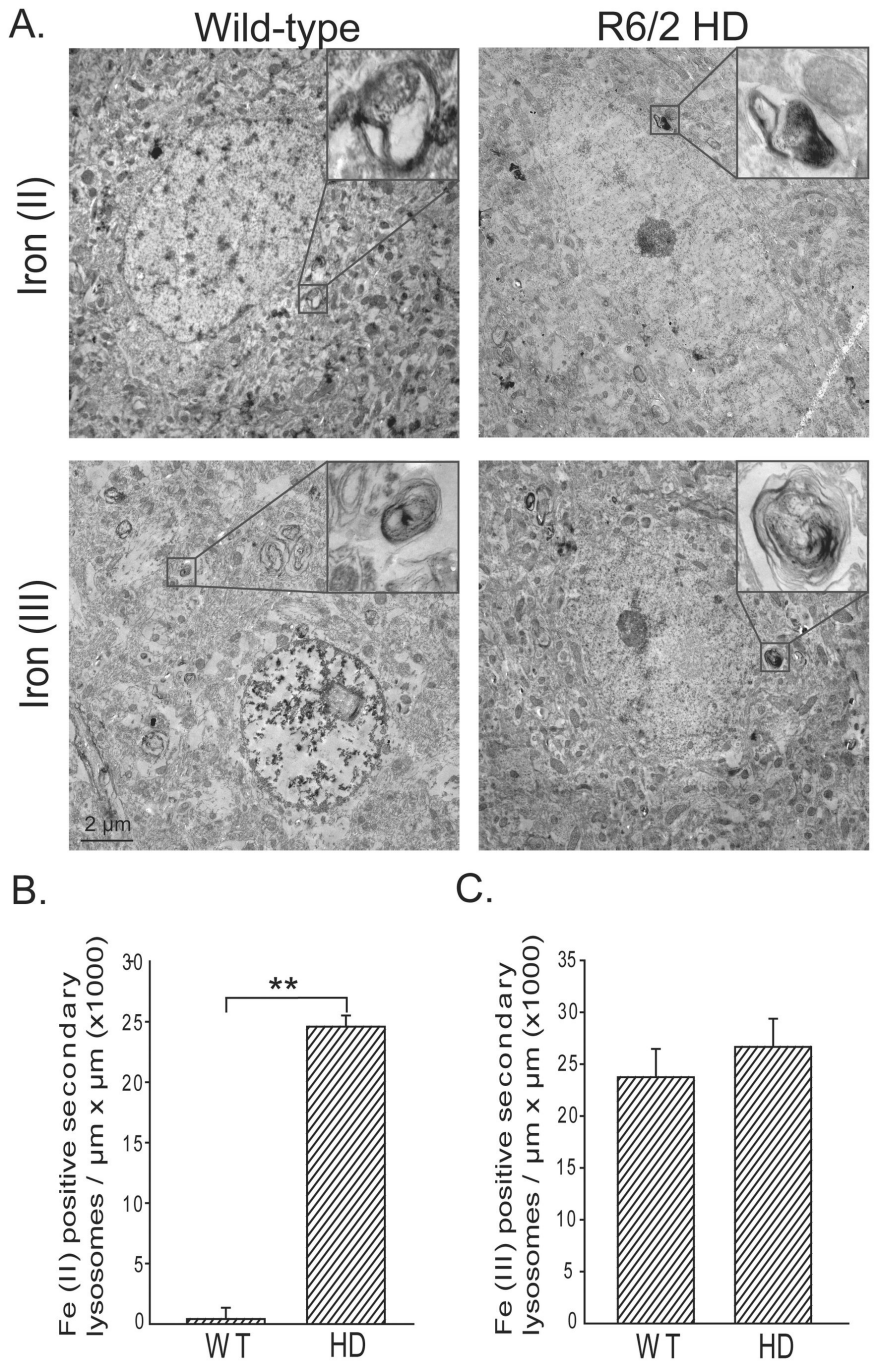
Iron (II) accumulates in R6/2 HD striatal neurons. Iron (II) and iron (III) were determined by a modification of the perfusion Turnbull’s and Perl’s iron stains (see methods). A. Electron photomicrographs show striatal neurons with foci of iron (II) or (III) staining in membrane-bound structures consistent with secondary lysosomes. Quantification of iron (II) (B) and iron (III) (C) staining reveals significantly elevated iron (II) while iron (III) is unaltered. P-value: **< 0.01, n=2 and 5 neurons / mouse .

To determine the subcellular location of iron accumulation in HD neurons we used perfusion iron staining methods for ferric (III) and ferrous (II) iron that also allowed combination with ultra-structural analysis (Figure **2**). Specifically, the Turnbull’s method was used to detect ferrous (II) iron accessible to ferricyanide and unmodified Perl’s method detected ferric (III) iron accessible to ferrocyanide. Additionally, we used modified Perl’s staining for total stainable iron (ferric and ferrous) on fixed brain sections combined with light microscopic analysis; this revealed increased staining in HD mice (Figure **S3A-D**). Turnbull’s staining revealed numerous areas of iron (II) staining in the striatal neuron cell body cytoplasm in rounded to ovoid structures that had complex membrane anatomy and ~0.5-0.7 µm in dimension (Figure **2A-B**). These were structurally consistent with secondary lysosomes [34], but attempts to provide more definitive subcellular identification by combining our approach with sub-cellular antibody markers were not successful. No difference in Perl’s iron (III) staining between wild-type and HD mouse striata was obtained (Figure **2A, 2C**) suggesting that the accumulation in total iron (Figures **S1** and **3A**) was present in the iron (II) form. Negative control brains not perfused with Perl’s or Turnbull’s solution did not have these electron dense deposits (not shown). These iron staining methods are reported not to detect heme iron. To confirm the specificity of iron these changes, a non-heme iron assay showed increases in striata and cortex of R6/2 HD brain (Figure **S4**).

**Figure 3:**
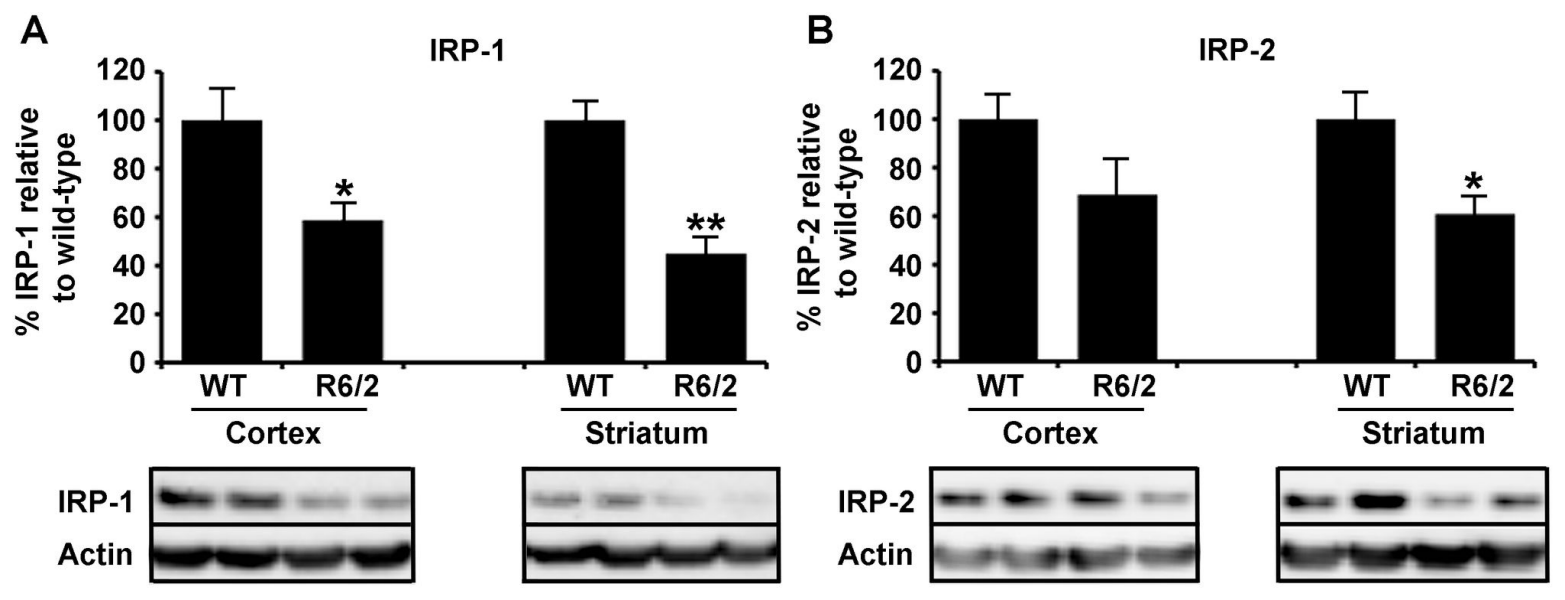
Decreased expression of iron-response proteins IRP1 and IRP2 in R6/2 HD mice. **A**. IRP-1 protein expression in cortical and striatal separated brain tissue was significantly decreased in 12-week-old R6/2 mice compared to age-matched wild-type littermate controls. **B**. To a lesser extent IRP-2 expression was also decreased in HD model tissue from the same mice but did not reach significance in cortical regions. P-values: *<0.05, **< 0.01, n=5.

Intra-neuronal iron homeostasis is tightly controlled by IRP-1 and IRP-2 via the labile iron pool (LIP). These proteins translationally regulate the expression of proteins involved in the transport of iron via neuronal uptake, storage or efflux [35]. In response to an increase in LIP, IRP-1 and 2 expression is reduced and accordingly IRP-1, and to a lesser extend IRP-2, are reduced in striatal and cortical tissue from R6/2 HD mice (Figure **3**).

**Figure 4:**
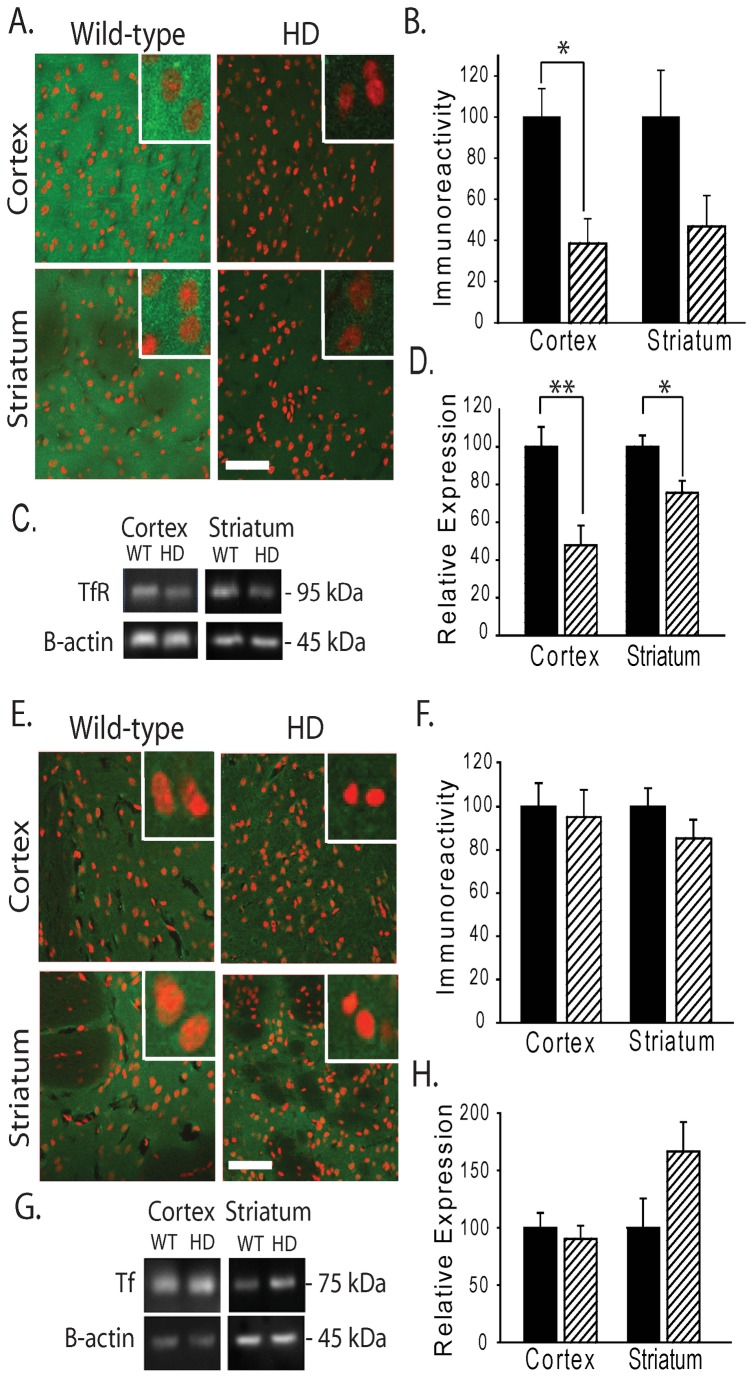
Decreased expression of transferrin receptor (TfR) but not transferrin (Tf) in R6/2 HD mice. Analysis of TfR (A-D) and Tf (E-H) protein expression in brain. **A**. Representative images indicate decreased TfR immunoreactivity in HD TG cortex and striatum as compared to wild-type mice at 12-weeks of age. (Red, DRAQ5 stain; Green, TfR). Bar = 50 μm. Inserts = 20 μm. **B**. Quantification of neuronal cell body TfR immunoreactivity in cortex and striatum of wild-type and R6/2 mice. **C**-**D**. Western blot analysis of TfR detects a band migrating at ~95 kDa consistent with TfR (**C**); this band is significantly decreased in HD as compared to wild-type mice in cortex and striatum (**D**). **E**. Representative images of Tf immunofluorescence in HD and wild-type litter-mate mice. **F**. Quantification of Tf immunoreactivity. **G**-**H**. Western blot analysis of Tf detects a band migrating at ~75 kDa consistent with Tf (**G**); there is no change in protein levels in HD mice (**H**). P-values: *<0.05, **< 0.01, n=3-4 for immunofluorescence analyses and n=9-10 for Western blot analyses.

Iron uptake is predominantly mediated via the transferrin receptor / transferrin system but also through other general divalent metal transporters such as the divalent metal transporter 1 system (DMT1). In accordance to the changes in IRP levels, TfR expression was decreased in cortex and striatum (Figures **4A-D** and **S3F**) suggesting a homeostatic IRP response to reduce further iron uptake caused by elevated labile iron. The iron carrying protein transferrin, present extra-cellularly and not directly regulated by neuronal IRP was found to be unaltered (Figure **4E-F**).

**Figure 5:**
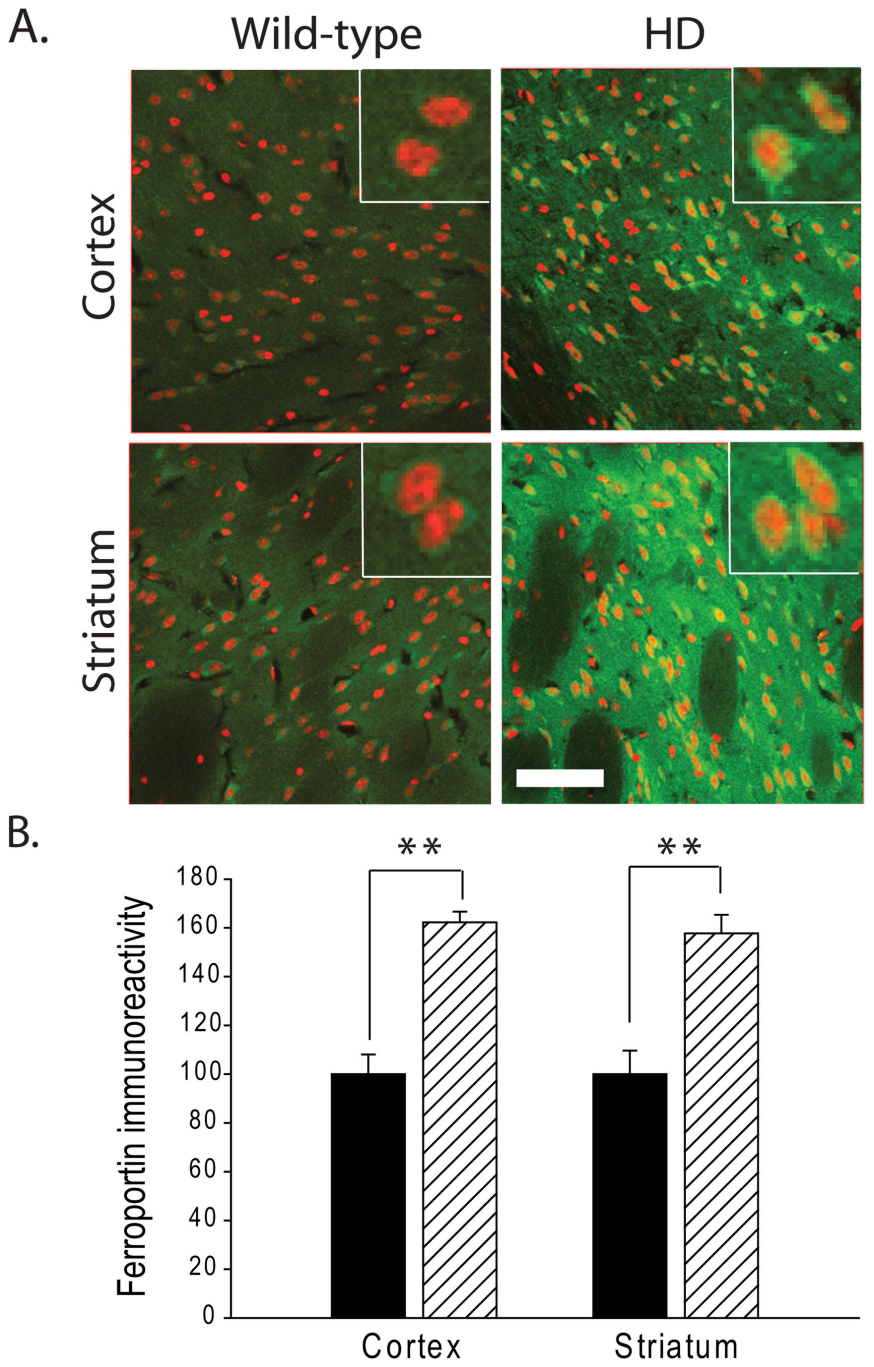
Increased expression of ferroportin (FPN) in R6/2 HD mice. **A**. FPN immunoreactivity is increased in HD mouse cortical and striatal neurons as compared to wild-type mice at 12 weeks (red, DRAQ5 stain; green, FPN). Bar = 50 μm. Inserts = 20 μm. **B**. Quantification of A. FPN immunoreactivity is increased ~0.6 fold in both cortex and striatum of HD mice as compared to WT litter mate mice. P-value: **< 0.01, n=3-4.

Ferroportin is currently the only cell iron export channel protein [26]. Quantifying the levels of Fpn by immunofluorescence staining found a significant increase in fluorescence signal in R6/2 HD mice (Figure **5**). To demonstrate specificity of the immunofluorescence staining for Fpn control staining of brain sections using a blocking peptide inhibited about ~90% immunoreactivity (not shown). Elevated ferroportin expression was again consistent with a homeostatic upregulation as part of an attempt to remove excess labile cytosolic iron. 

**Figure 6:**
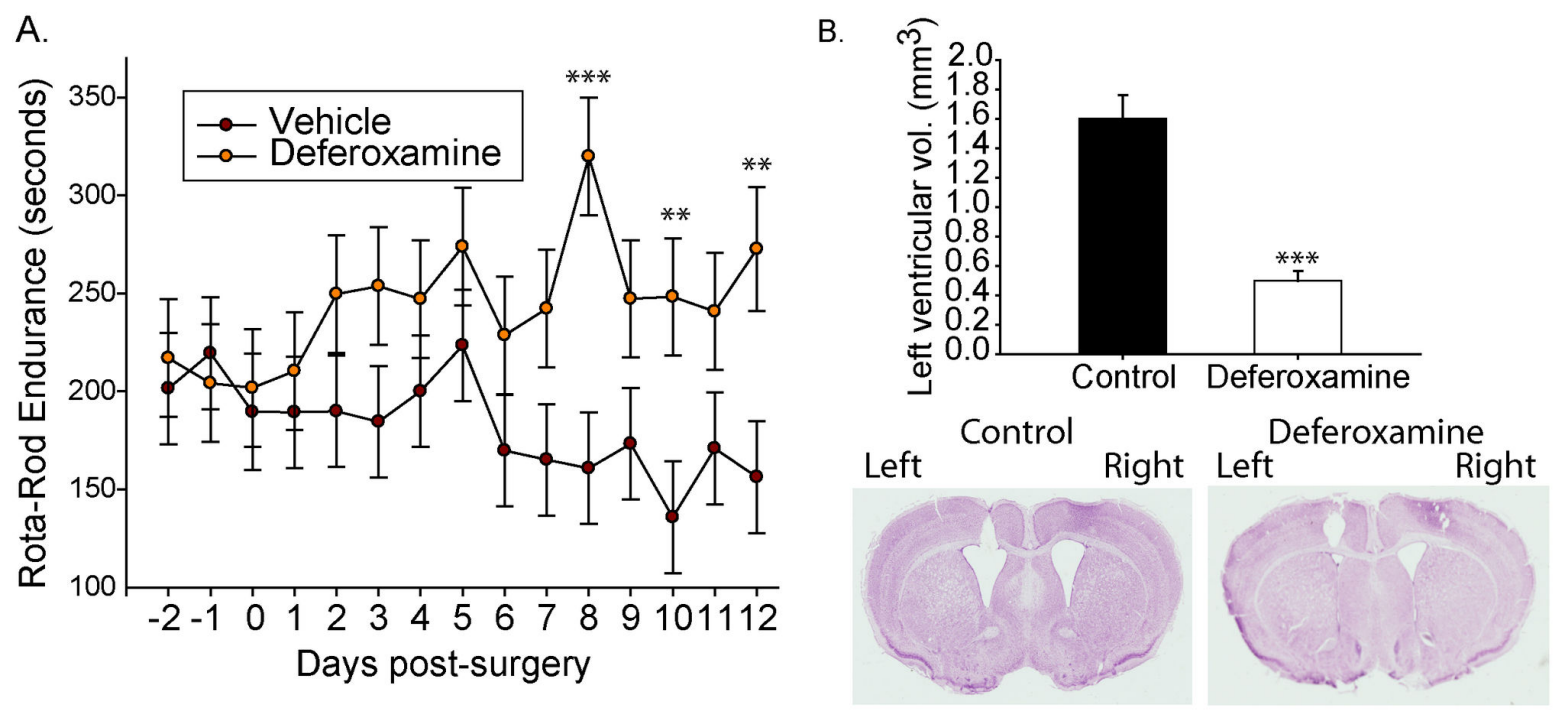
Intra-ventricular delivery of the iron chelator deferoxamine provides protection in R6/2 HD mice. Deferoxamine was delivered into the left ventricle of 6-week-old R6/2 HD mice at a dose-rate of 1-nmole / hour. Mice were analyzed daily before and after surgery for Rota-rod performance. **A**. Deferoxamine treated mice improve on Rota-Rod performance while vehicle-treated mice decline. **B**. Ventricular volumes are significantly lower in deferoxamine mice on the treated-side. P-values: **<0.01, ***<0.001: n=7.

To test whether iron accumulation within the suggested secondary lysosomal compartment was a compounding factor to neuronal stress or damage in the R6/2 HD model iron chelation was investigated. Deferoxamine is a potent iron chelator that is delivered into the endocytic pathway and is reported to reach secondary lysosomes [36]. As blood-brain-barrier penetration is very poor, we delivered this drug into the left ventricle of 6-week old R6/2 HD mice using an osmotic pump. Deferoxamine-treated HD mice demonstrated gradual improvement in Rota-rod endurance over the 2-week dosing period in contrast to vehicle-treated mice that continued to show the typical behavioral deterioration known to occur within this model (Figure **6A**). Further, quantitative pathology identified that deferoxamine-treated mice had significantly smaller lateral ventricles on the treated side (Figure **6B**).

## Discussion

A major goal of this study was to further understand where iron accumulates in HD brain. Iron accumulation in HD brain could be disease potentiating, for example, if unbound iron is catalyzing the generation of reactive oxygen species [37]. Alternatively, increased iron could result from elevated expression of protective iron proteins (such as the heme protein neuroglobin [38]). Therefore determining the cell and subcellular locations of elevated brain iron in HD, as well as the molecular associations and oxidation state is required to fully understand pathophysiology. We demonstrate that brain iron accumulation in R6/2 mouse HD occurs, at least partly, in neurons as determined by x-ray fluorescence (XRF) and electron microscopy (Figures **1**-**2**). While XRF measures the distribution of all forms of iron [39] our ultra-structural approach detects non-heme iron which could include iron that is free or present within ferritin or iron-sulfur proteins. Therefore, the combined finding of peri-nuclear iron foci in striatal neurons by XRF (Figure **1**) and membrane-bound structures with elevated iron (II) staining in the same location (Figure **2**) is consistent with the accumulation of non-heme iron in these structures. Based on size, membrane morphology and lack of internal cristae these structures are consistent with secondary lysosomes [40]. While we cannot fully exclude the possibility that they are late-endosomes, we believe this to be less likely. Unfortunately, the severe acidic conditions required for Turnbull’s staining destroyed the epitopes required to conclusively identify antibody markers of secondary lysosomes.

Increased macroautophagy is an important feature of HD [41]. While we have previously reported an approximate 2-2.5 fold increase in expression of the LAMP1 lysosomal marker protein in R6/2 HD mouse brain [29], here we found a >25 fold increase in iron (II) positive membrane bound vesicles. Therefore, if the iron (II) identified is within secondary lysosomes, our findings supporting a true increase in iron (II) content within this organelle rather than a secondary effect of increased secondary lysosome abundance. Iron (II) is potentially toxic due to its ability to freely generate oxygen radicals in an aerobic environment. This occurs when compounds such as ascorbate and glutathione reduce iron from the 3+ to the 2+ oxidation state. Iron (II) can then become re-oxidized and in the process donate an electron to hydrogen peroxide generating the highly toxic hydroxyl radical that is able to damage a variety of macromolecules including lipids and proteins. Therefore, our findings are compatible with endosomal and / or lysosomal iron-induced oxidative stress as has been reported previously in another model system [36]. Importantly, the distribution of iron we found is different to that reported for mutant huntingtin aggregates in this mouse line [42] consistent with our prior finding that iron does not interact with N-terminal fragments of huntingtin [20].

Simmons et al [43] reported iron accumulation in human brain HD microglial cells using the Perl’s method with light microscopy; R6/2 HD microglia did not have detectably elevated iron even though microglial ferritin was increased [43]. Human HD is characterized by dramatic neuronal loss especially in striatum, but also cerebral cortex [44]. R6/2 HD mice at 12-weeks of age have brain weights and striatal volumes that are ~20% less than wild-type litter-mate mice [45]. Striatal neuronal cell bodies are also smaller [45] than wild-type counterparts and a small percentage exhibit a non-apoptotic degenerative morphology [46]; however, striatal neuronal loss in R6/2 HD mice has not been reported. Therefore, the discrepancy between the finding of elevated microglial iron in human HD [43] and our findings of elevated neuronal iron (Figure **1**) could be explained by the lack of dramatic neuronal loss in R6/2 HD mice. In human HD, microglia may phagocytose sick or dying neurons contributing to iron accumulation [47,48]. Alternatively, neuronal loss in human HD may incite inflammation which is able to upregulate iron uptake pathways in microglial cells [49].

Brain iron is carefully regulated by a series of proteins important in cell uptake, export and transport. Expression level of most of these proteins is regulated by iron status through control of IRPs on their respective iron-response element (IRE) mRNA hairpins (present in untranslated regions of mRNA) [50]. Through this system iron excess results in compensatory decreased levels of iron uptake proteins and increased export proteins. Conversely, iron deficiency results in compensatory up-regulation of cell iron uptake and decreased export proteins. Our protein expression studies, focusing on neurons, showed decreased expression of IRPs (Figure **3**) with a corresponding decrease in the transferrin receptor iron uptake protein and increased expression of the iron export protein ferroportin (Figures **4**-**5**). We interpret these findings to indicate a compensatory response to iron stress occurring in HD brain. Interestingly, it has previously been reported that there are changes in transferrin receptor re-cycling in HD cell lines which could also affect iron uptake [51]. We have reported previously that APP levels are decreased in R6/2 HD brain [20]. Of note APP also contains an IRE [52] and has more recently been identified as being involved in the facilitation of iron export from neurons through its interaction with Fpn [22]. As it would be expected that APP expression would be increased in high LIP, it could be proposed that the increased neuronal iron accumulation in mouse HD neurons, as we report here, may be a result of aberrant neuronal iron export caused by an insufficient APP iron-export mechanism.

Iron in HD neurons could contribute to degenerative processes by promoting subcellular oxidative damage at sites of accumulation. For proof of concept, we tested the effect of deferoxamine, a hexadentate highly selective iron chelator which upon binding iron completely silences its redox activity [53] and is reported to be taken up by fluid phase endocytosis into lysosomes [47]. By intra-thecal infusion a protective effect of deferoxamine was observed in R6/2 HD mice even after only 2-weeks of treatment (Figure **6**). While the exact mechanism of protection and cellular / subcellular sites of action has currently not been identified this result supports the presence of iron stress / toxicity in HD brain and is consistent with an endo-lysosomal site of injury. Consistent with our findings Firdaus et al [54] reported decreased oxidation of oxidative stress probes in deferoxamine treated HD cell lines. Unfortunately, peripheral deferoxamine delivery is unlikely to be feasible in human HD due to its lack of blood brain penetrability and high chance of causing systemic iron deficiency. Alternative classes of brain permeable low affinity metal-protein attenuating agents are available (e.g. the 8-hydroxy-quinolones). Both clioquinol and PBT2 have shown protective effects in yeast, worm and mouse HD models [55-57] and thus may in part be targeting our proposed pathway. 

The iron localization studies, iron homeostatic protein responses and protective effects of iron chelation with deferoxamine support a potentiating role of altered iron homeostasis in mouse HD. While a recent human HD brain imaging study showed no correlation between MRI-measured brain iron elevation and the level of atrophy [58], iron within the brain is present in many forms and MRI-visible iron is thought to predominantly only represent ferritin-bound iron (III) [59]. Thus, MRI-measured iron elevations in human HD brain may only identify the increased microglial ferritin iron as has previously been reported [43]. Further, ferritin may provide protection from iron-induced oxidative stress, as occurs in a mouse model of Parkinson’s disease [13]. Taken together our findings indicate that neurons are an important site of toxic iron (II) accumulation in HD. Further investigation of the nature of iron dysregulation may lead to new approaches to therapeutically manipulate brain iron in HD. 

## Supporting Information

Figure S1 **Time course analysis of behavioral changes and brain iron elevation in R6/2 HD mice.** **A**-**D**. Studies in R6/2 HD mice. **A**-**B**. Time-course of total brain regional iron concentrations as measured by ICP-MS. n=10-14, A. Cortical iron levels are significantly increased at 8 and 12-weeks of age in HD mice. Interaction p<0.001, B. Striatal iron levels are significantly increased across all ages [main effect p-value for genotype <0.0001]. There is not a significant genotype x age interaction. Pair-wise post-hoc t-test reveals significant elevation of iron at 12-weeks. **C**. Deficits in Rota-rod performance are first detected at 6 weeks of age. n=10-12, D. Significant decrease in spontaneous wheel activity is first detected at 8-weeks of age. n=10-12. Asterisks indicate levels of significance: *< 0.05, **< 0.01, ***< 0.001. Bars: black bars = wild-type; cross-hatched = R6/2.
(EPS)

Figure S2 **Time course analysis of behavioral changes and brain iron elevation in N171-82Q HD mice.** **A**-**C**. Studies in N171-82Q HD mice. **A**-**B**. Time-course of total brain regional iron concentrations as measured by ICP-MS. n=15, **A**. Cortical iron levels are significantly increased at 12 and 20-weeks of age in HD mice. **B**. Striatal iron levels are not different between wild-type and HD mice. **C**. Deficits in Rota-rod performance are first detected at 20-weeks of age. Interaction p=0.003, n=16-19. Bars: black bars = wild-type; cross-hatched = R6/2.
(TIF)

Figure S3 **Supportive evidence of intracellular iron increased in R6/2 HD mouse cortex and striatum.** **A**-**D**.
Modified Perl’s staining on total iron in cortical (A-B) and striatal (C-D) tissue from R6/2 HD mice (B-D) is increased compared to wild-type littermate controls (A-C) at 12 weeks of age. **E**. Quantitation with representative blots of ferritin (Ft) expression in cortical and striatal tissue from R6/2 HD mice and wild-type littermate controls at 12-weeks of age. Ft was significantly increased in R6/2 cortex but not in the striatum. F. Quantitation with representative blots of TfR expression illustrating the same trend as observed in Figure 4, using an antibody with an alternative epitope to the protein. TfR expression in cortical and striatal tissue from R6/2 HD mice at 12-weeks of age is significantly decreased in R6/2 cortex compared to wild-type littermate controls. P-values: *<0.05, **< 0.01, n=5.
(TIF)

Figure S4 **Non-heme iron is increased in R6/2 HD mouse brain.** R6/2 HD and wild-type litter-mate mice were sacrificed at 12-weeks of age. Non-heme iron levels (see methods) were significantly elevated in striata and cortices of HD mice. P-value: ***< 0.001, n=10.
(EPS)

Methods S1 **Supplementary methods for x-ray fluorescence and perfusion iron staining.**
(DOCX)
