# Correction: Iron Accumulates in Huntington’s Disease Neurons: Protection by Deferoxamine 

**DOI:** 10.1371/annotation/67f555f5-35b7-4468-8bab-26d518942803

**Published:** 2013-11-13

**Authors:** Jianfang Chen, Eileen Marks, Barry Lai, Zhaojie Zhang, James A. Duce, Linh Q. Lam, Irene Volitakis, Ashley I. Bush, Steven Hersch, Jonathan H. Fox

The second and last authors were incorrectly indicated as having contributed equally to the article.

In addition, the first author, Jianfeng Chen, was not correctly indicated as having contributed equally to the article with the second author, Eileen Marks. 

