# Supplementary figures and images for: Iron Accumulates in Huntington’s Disease Neurons: Protection by Deferoxamine
Source: PLoS One. 2013 Oct 11;8(10):e77023. doi: 10.1371/journal.pone.0077023 (PMC3795666; doi:10.1371/journal.pone.0077023)

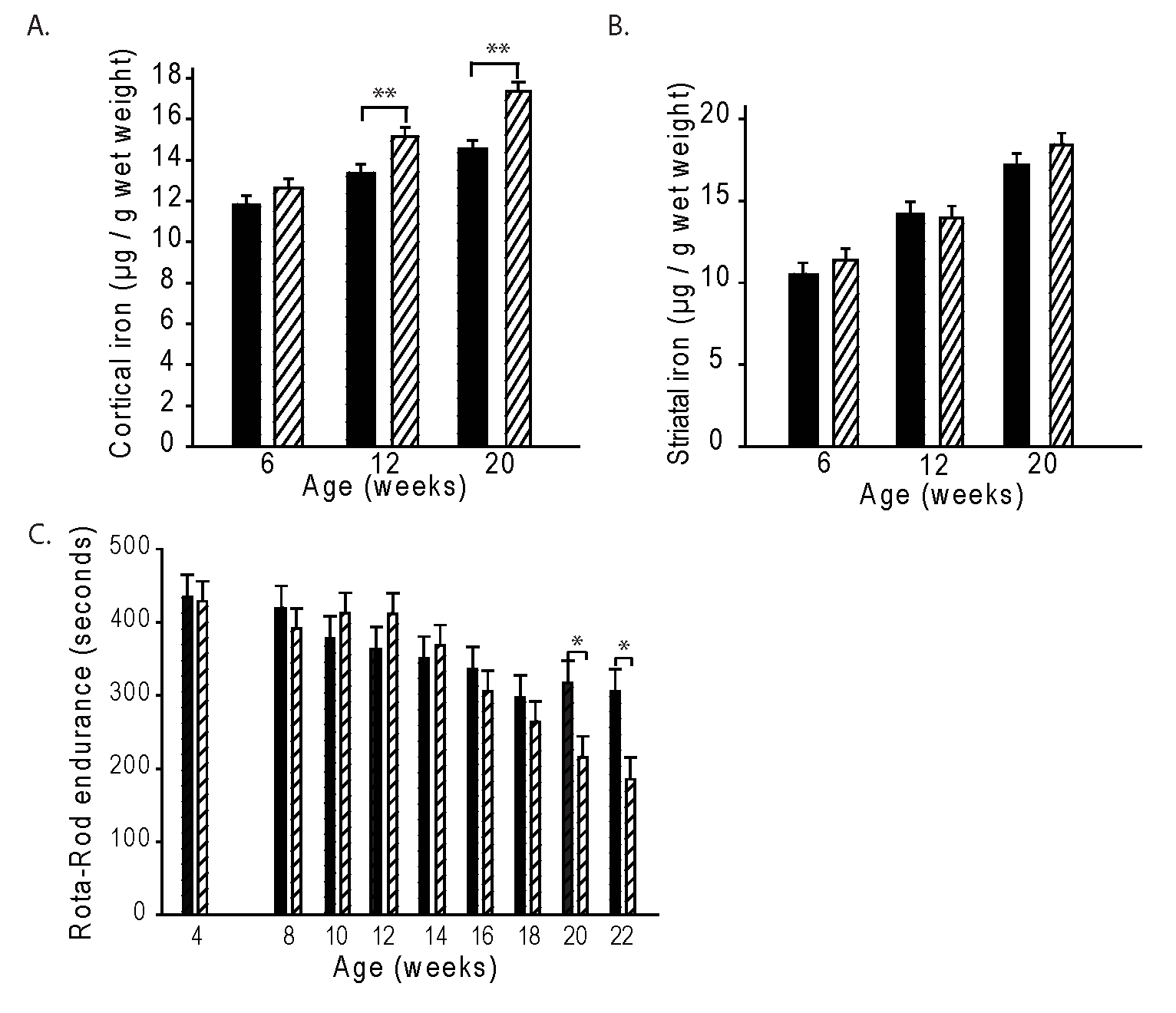

Supplement: Figure S2 — Time course analysis of behavioral changes and brain iron elevation in N171-82Q HD mice. A-C. Studies in N171-82Q HD mice. A-B. Time-course of total brain regional iron concentrations as measured by ICP-MS. n=15, A. Cortical iron levels are significantly increased at 12 and 20-weeks of age in HD mice. B. Striatal iron levels are not different between wild-type and HD mice. C. Deficits in Rota-rod performance are first detected at 20-weeks of age. Interaction p=0.003, n=16-19. Bars: black bars = wild-type; cross-hatched = R6/2. (TIF) [file pone.0077023.s002.tif]

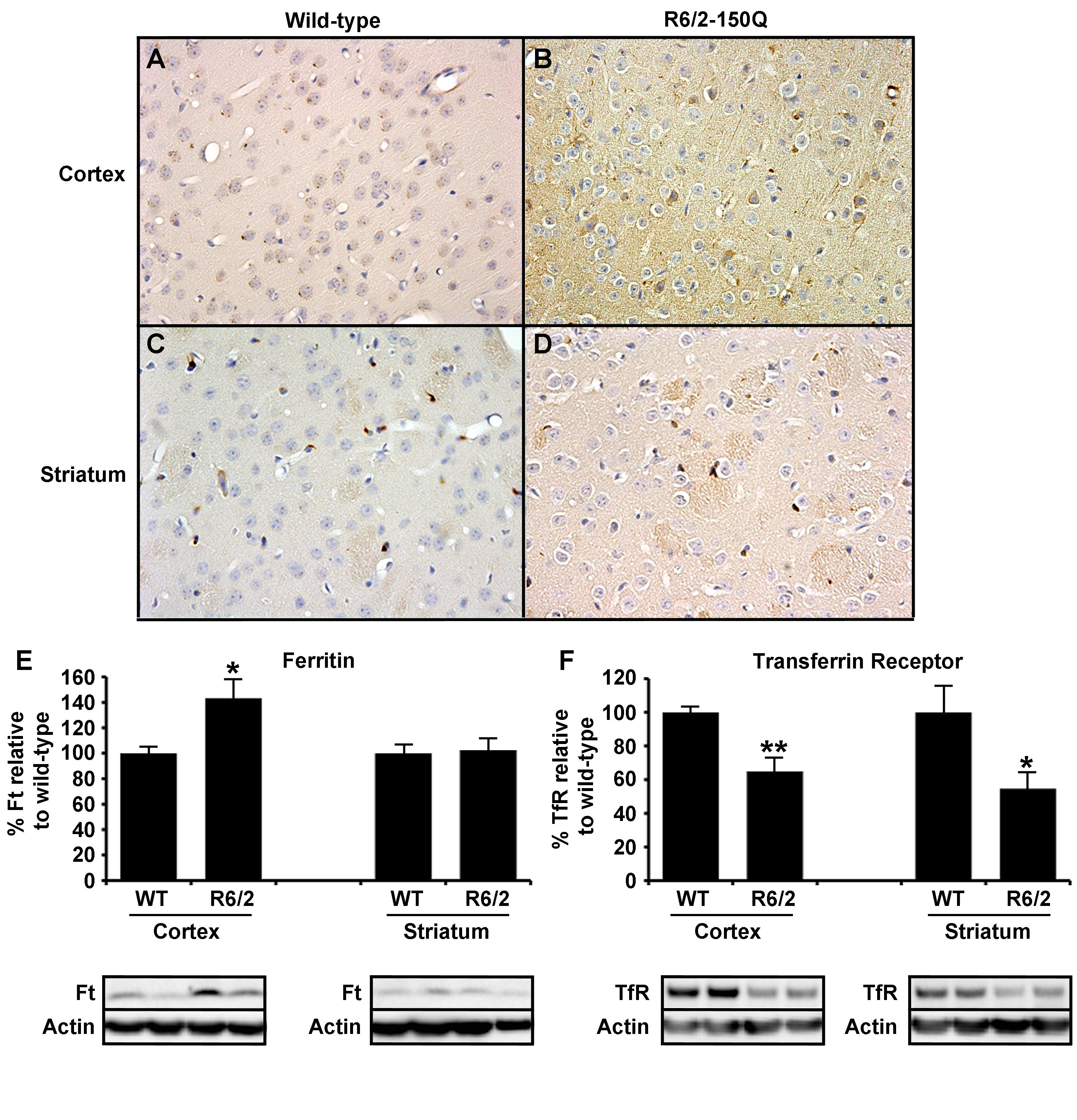

Supplement: Figure S3 — Supportive evidence of intracellular iron increased in R6/2 HD mouse cortex and striatum. A-D. Modified Perl’s staining on total iron in cortical (A-B) and striatal (C-D) tissue from R6/2 HD mice (B-D) is increased compared to wild-type littermate controls (A-C) at 12 weeks of age. E. Quantitation with representative blots of ferritin (Ft) expression in cortical and striatal tissue from R6/2 HD mice and wild-type littermate controls at 12-weeks of age. Ft was significantly increased in R6/2 cortex but not in the striatum. F. Quantitation with representative blots of TfR expression illustrating the same trend as observed in Figure 4, using an antibody with an alternative epitope to the protein. TfR expression in cortical and striatal tissue from R6/2 HD mice at 12-weeks of age is significantly decreased in R6/2 cortex compared to wild-type littermate controls. P-values: *<0.05, **< 0.01, n=5. (TIF) [file pone.0077023.s003.tif]
