# Supplementary material for: Iron Accumulates in Huntington’s Disease Neurons: Protection by Deferoxamine
Source: PLoS One. 2013 Oct 11;8(10):e77023. doi: 10.1371/journal.pone.0077023 (PMC3795666; doi:10.1371/journal.pone.0077023)
Supplement: Methods S1 — Supplementary methods for x-ray fluorescence and perfusion iron staining. (DOCX) [file pone.0077023.s005.docx]

**Supplementary methods**

**Synchrotron X-ray Fluorescence Microscopy (micro-XRF):** Scanning X-ray fluorescence microscopy was performed at the 2-ID-D Station of the Advanced Photon Source at Argonne National Laboratory (IL, USA) [1]. The source was a 3.3-cm-period undulator, and x-rays were monochromatized by a double-crystal Si (111) monochromator. A Fresnel zone plate focused the x-ray beam to a spot size of 0.3 (h) x 0.2 (v) µm^2^ on the sample. The flux at the focal spot was measured to be ~ 4x10^9^ photons / second using an ionization chamber in place of the sample [2]. The specimen was placed in a helium environment and mounted on a X-Y translation stage at 75º to the incident beam. An energy-dispersive silicon drift detector (Hitachi High-Tech Science America) was used to collect x-ray fluorescence spectra from the sample while it was being scanned across the focus spot. In this way, spatial maps of many different elements were acquired simultaneously. An incident x-ray energy of 10 keV was used to excite K_α_ emission lines for elements from Si to Zn. At this energy x-rays penetrates biological specimens without significant absorption nor beam spreading; thus no specimen thinning is required and fluorescence maps of individual elements represent a 2D projection of the volumetric distribution within the specimen [3,4].

The specimen supported on a thin silicon nitride membrane was placed onto a kinematic specimen holder suitable for both optical and X-ray fluorescence microscopy. The specimen was first examined under a visible light microscope (Leica DMXRE) and selected areas of interest were located relative to reference points of the silicon nitride membrane using a high resolution motorized x/y stage (Ludl Bioprecision). Coordinates were recorded and were used to precisely locate the target area once the specimen was transferred to the x-ray microprobe.

Spectral analysis was performed using the program MAPS [5]. The spectrum at each pixel was individually fitted to remove overlaps between adjacent K_α_ emission lines in order to achieve more accurate quantification. Conversion of elemental fluorescence intensities to areal densities in μg / cm^2^ was performed by comparing x-ray fluorescence intensities with those from thin film standards XRM-1832 and XRM-1833 (NIST, Gaithersburg, MD).

Addition review articles on micro-XRF applications are available [6,7].

**Perfusion-Perl’s and Turnbull’s methods for subcellular localization of iron in HD BRAIN**

Overview of approach: Tissue iron stains reveal forms of iron that are available to react with the chemical stain. This includes iron that is unbound and loosely bound to protein but not iron tightly bound to heme and iron proteins. As we think that excess loosely bound iron may promote oxidative stress our goal was to identify the subcellular location within neurons of iron II and / or III accumulation. Post-fixation staining methods have the disadvantage of not staining the ionic form of iron (II or III) present in the living tissue. Iron (II) will be largely oxidized to iron (III) after life. Therefore, to accurately stain these forms of labile iron we perfused with a fixative containing the iron reactive compound.

Chemistry of iron detection: Perl’s stain contains 1% potassium ferrocyanide which releases ferrocyanide ion and reacts with iron (III) which is liberated in acidic solution to form Prussian blue. Turnbull’s stain contains potassium ferricyanide which releases ferricyanide ion and reacts with iron (II) which is liberated in acidic solutions to form Turnbull blue. Prussian blue and Turnbull blue can be intensified with diaminobenzadine.

Solutions:

**Buffer A**: 0.9% (w/v) saline and 0.005 M phosphate buffer, pH 7.4.

**Buffer B**: 0.9% (w/v) saline and 0.01 M phosphate buffer, pH 7.4.

**Pre-fixative**: 0.5% (v/v) glutaraldehyde and 4% (w/v) paraformaldehyde in buffer A.

**Perl’s fixative**: 1% potassium ferrocyanide and 2% paraformaldehyde in water, pH 0.8-1.0.

**Turnbull’s fixative**: 1% potassium ferricyanide and 2% paraformaldehyde in water, pH 0.8-1.0.

**Post-fixative**: 0.5% glutaraldehyde in buffer A, pH 5.5-6.0.

Fixatives were made fresh on the day of use. Immediately before perfusion, the desired pH of the fixative was obtained using concentrated HCl.

Protocol: We modified a described procedure for staining labile iron II and III in brain tissue [8]. We decreased the perfusion rate from 20 ml/minute to 10 ml/minute and kept heparinized saline and pre-fixative at 37 °C to prevent blood vessel constriction. We also added nitrite as a vasodilator to promote even tissue perfusion. We made Perl’s and Turnbull’s fixative solutions in water instead of saline to enable physiologic osmolarity.

Mice were deeply anesthetized and perfused through the left ventricle with freshly prepared 37°C heparinized buffer A containing 0.1% (w/v) sodium nitrite for 6 minutes at 10 ml/minute. Mice were then immediately perfused with 37°C pre-fixative for 20 minutes at 10 ml/minute, followed by Perl’s, Turnbull’s or pre-fixative (this is a control for background staining from DAB) for 30 minutes at 10 ml/minute. Finally, mice were perfused with 0.9% saline for 3 minutes at 10 ml/minute to flush out excess fixative. Perfused mice were placed in sealed plastic bags and stored at 4°C for 1 hour. Brains were dissected and the whole brains immersed in post-fixative overnight at 4°C. The following day brains were sectioned at 40 μm in buffer A using a vibratome then washed and stored in 0.9 % saline at 4°C. Sections were treated with 0.3% (v/v) H_2_O_2_ and 0.065% (w/v) sodium azide (as a bacteriostat) in buffer A for 15 minutes, followed by 5 minutes wash with buffer A. Then sections were incubated in DAB solution (0.025% diaminobenzidine in buffer B) for 10 minutes. Finally, sections were treated with DAB solution containing 0.005% (v/v) H_2_O_2_ for 20 minutes for Perl’s and 40 minutes for Turnbull’s, followed by wash with buffer B. [If light microscopy sections are needed, sections can be mounted onto slides and dried overnight at room temperature then washed in distilled water for 10 seconds and dehydrated with a graded ethanol series (70%, 95%, 95%, 100%, 100%, 3 minutes for each) and xylene twice 3 minutes prior to cover slipping].

Striatum from sections for electron microscopy were cut into squares less than 1 mm in greatest dimension with the aid of a dissecting scope and post-fixed in 1% osmium-tetroxide in 0.1M phosphate buffer for 60 minutes. After washing with distilled water, striata were dehydrated with a graded ethanol series (30%, 50%, 75%, 85%, 95% and 100% three times, 15 minutes for each). Then sections were treated with different ratios of propylene oxide and Epon mixture (3:1 overnight; 1:1 for 4 hours; 1:3 for 4 hours; 100% Epon three times: overnight, 3 hours and 3 hours). Finally sections were embedded in Epon for >24 hours at 60˚C. Tissue blocks were sectioned at 50 nm with a diamond knife on an MRC-MTXL ultramicrotome. Thin sections were collected on 200 mesh copper grids and stained with 2% uranyl acetate for 30 minutes and lead citrate for 10 seconds.

Digital images were obtained using a Hitachi 7000 transmission electron microscope. For each mouse, five neurons (for perfusion-Perl’s and Turnbull’s) were identified at low magnification (3K). Neurons were identified based upon nuclear size. For perfusion-Perl’s and Turnbull’s methods, iron-positive secondary lysosomes were identified at high magnification (30K). The density of these iron-positive structures was quantified by counting and normalizing to the area of neuronal cytoplasm evaluated.

**Supplementary references**

1. Cai Z, Lai, B., Yun, W., Ilinski, P., Legnini, D., Maser, J., and Rodrigues, W. A Hard X-Ray Scanning Microprobe for Fluorescence Imaging and Microdiffraction at the Advanced Photon Source. Am. Inst. of Physics Conf. Proc.; 2000. pp. 472-477.

2. Yun W, Lai, B., Cai, Z., Maser, J., Legnini, D., Gluskin, E., Chen, Z., Krasnoperova, A., Valdimirsky, Y., Cerrina, F., Di Fabrizio, E., Gentili, M. (1999) Nanometer Focusing of Hard X-Rays by Phase Zone Plates. Rev Sci Instrum 70: 2238-2241.

3. Twining BS, Baines SB, Fisher NS, Maser J, Vogt S, et al. (2003) Quantifying trace elements in individual aquatic protist cells with a synchrotron X-ray fluorescence microprobe. Anal Chem 75: 3806-3816.

4. Kemner KM, Kelly SD, Lai B, Maser J, O'Loughlin E J, et al. (2004) Elemental and redox analysis of single bacterial cells by x-ray microbeam analysis. Science 306: 686-687.

5. Vogt S (2003) MAPS: A set of software tools for analysis and visualization of 3D X-ray fluorescence data sets. J Phys 104: 635-638.

6. Fahrni CJ (2007) Biological applications of X-ray fluorescence microscopy: exploring the subcellular topography and speciation of transition metals. Curr Opin Chem Biol 11: 121-127.

7. Paunesku T, Vogt S, Maser J, Lai B, Woloschak G (2006) X-ray fluorescence microprobe imaging in biology and medicine. J Cell Biochem 99: 1489-1502.

8. Meguro R, Asano Y, Odagiri S, Li C, Shoumura K (2008) Cellular and subcellular localizations of nonheme ferric and ferrous iron in the rat brain: a light and electron microscopic study by the perfusion-Perls and -Turnbull methods. Arch Histol Cytol 71: 205-222.
